# The Formation of *β*-Strand Nine (*β*_9_) in the Folding and Insertion of BamA from an Unfolded Form into Lipid Bilayers

**DOI:** 10.3390/membranes13020247

**Published:** 2023-02-19

**Authors:** Sascha Herwig, Jörg H. Kleinschmidt

**Affiliations:** 1 Institut für Biologie, FB 10 Mathematik und Naturwissenschaften, Universität Kassel, Heinrich-Plett-Str. 40, D-34132 Kassel, Germany; 2 Center of Interdisciplinary Nanostructure Science and Technology, Universität Kassel, Heinrich-Plett-Str. 40, D-34132 Kassel, Germany

**Keywords:** outer membrane protein, protein folding, lipid bilayer, *β*-barrel, BamA, OmpA, cysteine scanning mutagenesis, site-directed fluorescence spectroscopy, BAM complex

## Abstract

Transmembrane proteins span lipid bilayer membranes and serve essential functions in all living cells. Membrane-inserted domains are of either α-helical or *β*-barrel structure. Despite their biological importance, the biophysical mechanisms of the folding and insertion of proteins into membranes are not well understood. While the relative composition of the secondary structure has been examined by circular dichroism spectroscopy in folding studies for several outer membrane proteins, it is currently not known how individual *β*-strands fold. Here, the folding and insertion of the *β*-barrel assembly machinery protein A (BamA) from the outer membrane of *Escherichia coli* into lipid bilayers were investigated, and the formation of strand nine (*β*_9_) of BamA was examined. Eight single-cysteine mutants of BamA were overexpressed and isolated in unfolded form in 8 M urea. In each of these mutants, one of the residues of strand *β*_9_, from R572 to V579, was replaced by a cysteine and labeled with the fluorophore IAEDANS for site-directed fluorescence spectroscopy. Upon urea-dilution, the mutants folded into the native structure and were inserted into lipid bilayers of dilauroylphosphatidylcholine, similar to wild-type BamA. An aqueous and a membrane-adsorbed folding intermediate of BamA could be identified by strong shifts in the intensity maxima of the IAEDANS fluorescence of the labeled mutants of BamA towards shorter wavelengths, even in the absence of lipid bilayers. The shifts were greatest for membrane-adsorbed mutants and smaller for the inserted, folded mutants or the aqueous intermediates. The spectra of the mutants V573C-, L575C-, G577C-, and V579C-BamA, facing the lipid bilayer, displayed stronger shifts than the spectra recorded for the mutants R572C-, N574C-, T576C-, and K578C-BamA, facing the *β*-barrel lumen, in both the membrane-adsorbed form and the folded, inserted form. This alternating pattern was neither observed for the IAEDANS spectra of the unfolded forms nor for the water-collapsed forms, indicating that strand *β*_9_ forms in a membrane-adsorbed folding intermediate of BamA. The combination of cysteine scanning mutagenesis and site-directed fluorescence labeling is shown to be a valuable tool in examining the local secondary structure formation of transmembrane proteins.

## 1. Introduction

**Figure 1:**
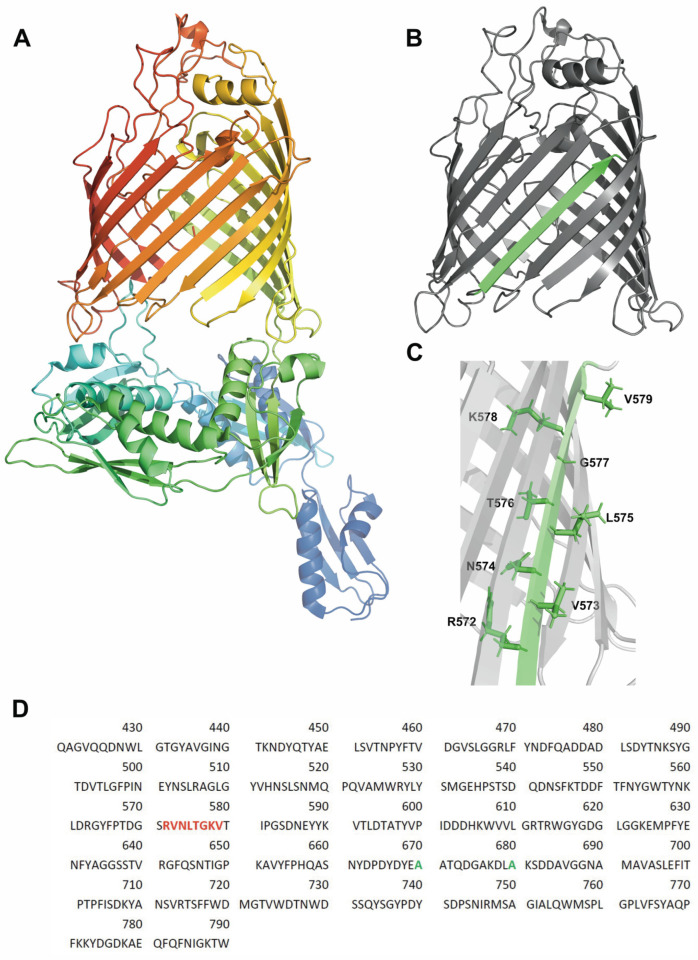
Local folding into the *β*-sheet secondary structure was investigated for strand nine (*β*_9_) of the barrel domain of BamA. (**A**) Cartoon of the crystal structure of BamA based on the structure coordinates from the BAM complex from *E. coli* (5D0O) [3]. wt-BamA comprises a 16-stranded *β*-barrel transmembrane domain and a periplasmic domain with a similar number of amino acid side chains. (**B**) Cartoon of the crystal structure of the transmembrane *β*-barrel of BamA. *β*_9_ is highlighted in green. All structures were drawn with Pymol [4]. (**C**) Side chains along the *β*_9_ of wt-BamA mutated to express and isolate eight single-cysteine mutants of BamA: R572C-, V573C-, N574C-, L575C-, T576C-, G577C-, K578C-, and V579C-BamA. These mutants were labeled with the fluorophore IAEDANS for site-directed fluorescence spectroscopy. (**D**) Amino acid sequence of the C-terminal *β*-barrel domain of BamA (residues 421 to 790) is shown. The amino acid residues of strand *β*_9_ are colored in red. All mutations were performed on a vector containing a *bamA* gene, in which the codons for the two native cysteines of BamA (green) were replaced by codons for alanine.

The integrity of cells or cell organelles is preserved by biological membranes. These consist of a lipid bilayer and either peripheral or integral membrane proteins. Integral membrane proteins, also called transmembrane proteins (TMPs), span the lipid bilayer and serve a wide range of different biological functions. In regard to the secondary structure of the lipid-facing domains, TMPs may fall into two categories: α-helical and *β*-barrel TMPs. The biophysical principles of how TMPs form their three-dimensional structure are not well understood. With the capsule transporter Wza as the only exception known to date, the TMPs of the outer membranes of Gram-negative bacteria are *β*-barrel TMPs. In these bacteria, *β*-barrel TMPs are translocated across the cytoplasmic membrane and the periplasm before they fold and insert into the outer membrane. Their transport is facilitated by chaperones in the periplasm and by the *β*-barrel assembly machinery (BAM-) complex of the outer membrane, an evolutionarily conserved complex also found in the outer membranes of the organelles of eukaryotic cells. In *E. coli,* this complex consists of the TMP BamA as the only transmembrane protein and four lipoproteins. BamA alone has been shown to facilitate the folding of outer membrane protein A (OmpA) into preformed lipid bilayers of dilauroylphosphatidylcholine (*di*C_12_PC) that serve as model membranes [1] by a mechanism that is not well understood but apparently requires the presence of both domains of BamA [1,2], as shown in Figure 1A.

*β*-barrel TMPs are known to unfold in solutions of chaotropic denaturants such as urea at high concentrations [5]. Upon the strong dilution of the denaturant, they can spontaneously fold into their functionally active structure in preformed detergent micelles [6,7,8], in lipid bilayers [9], or in amphipols [10]. In these model systems, the folding and insertion are slow but do not require any proteinaceous folding machinery; for reviews, see, e.g., [11,12,13].

Biophysical investigations on the mechanisms of unassisted folding and membrane insertion have been performed predominantly with the smaller eight-stranded *β*-barrel membrane proteins such as OmpA [14,15,16,17,18,19,20,21] or PagP [22,23,24]. In contrast, comparably fewer studies have been performed with the larger *β*-barrel membrane proteins such as the 16-stranded OmpF [8,25] or BamA [1], the 14-stranded OmpG [26] or FomA [10,27], or the 19-stranded VDAC [28]. Some of these outer membrane proteins such as OmpF do not fold well into lipid bilayers as model membranes either with low yields in the absence of detergents [25] or with very slow kinetics [29]. For others, folding kinetics are slow and time-consuming, as reported for FomA from *Fusobaterium nucleatum*, which spontaneously folded into *di*C_12_PC [27]. Reports of intermediates in the folding of these larger *β*-barrels have mostly focused on the formation of the OmpF dimers and trimers [8,25] and on aqueous forms that are formed in the absence of membranes. The circular dichroism spectra of the smaller OmpA indicated the formation of an aqueous intermediate. As observed by recording the CD signal as a function of time after the dilution of the denaturant [17,30], most of the *β*-sheet secondary structure forms upon the insertion of OmpA into the lipid bilayer. The time courses for *β*-sheet formation ranged from 10 min for folding into bilayers of didecanoyl phosphatidylcholine (*di*C_10_PC) to about 70 min for folding into *di*C_12_PC bilayers, i.e., dependent on the bilayer thickness [15,21].

In contrast to the eight-stranded *β*-barrel of OmpA, the larger *β*-barrel TMPs develop a significant *β*-sheet secondary structure already upon the dilution of the denaturant urea in the absence of any lipid or detergent, as reported for OmpF [25] or FomA [27] from bacteria or for the 19-stranded VDAC, human isoform 1 [28]. For this reason, it is important to examine the folding of the larger *β*-barrels in more detail. It was previously demonstrated that the 16-stranded *β*-barrel of BamA folds orientedly into preformed lipid bilayers (large unilamellar vesicles, LUVs) of *di*C_12_PC to high yields. Pre-inserted BamA, as well as its separately isolated transmembrane domain, catalyzed the folding and insertion of OmpA [1], as later confirmed by other studies [31]. However, the biophysical mechanism of the folding of BamA is not well understood.

Here, we examined the folding and insertion of BamA, which has a 16-stranded *β*-barrel transmembrane domain and forms monomers in lipid bilayers. We previously demonstrated that BamA folds orientedly into preformed lipid bilayers (LUVs) of *di*C_12_PC or of mixtures of *di*C_12_PC and dilauroylphosphatidylethanolamine (*di*C_12_PE) [1]. In the present study, we investigated whether membrane-bound folding intermediates of BamA can be identified, similar to those reported for OmpA [7,14,21,30,32,33]. As the formation of the structure of a single *β*-strand has not been reported to date, we used site-directed fluorescence spectroscopy on a range of mutants of BamA to examine the folding into the local secondary structure between residues 472 and 479, which comprise most of the strand *β*_9_ when BamA is fully folded. This strand was selected as one of the two central strands of the *β*-barrel domain of BamA. In folded *β*-barrels, residues along transmembrane *β*-strands alternate in their exposure to the lipid bilayer and to the barrel lumen (Figure 1). These investigations were performed after denaturant dilution in the absence and in the presence of lipid bilayers to determine whether the formation of strand *β*_9_ requires adsorption to the membrane.

## 2. Materials and Methods

### 2.1. Single-Cysteine Mutants of BamA

The *bamA* gene from *E. coli* was cloned between the *NcoI* and *BamHI* restriction sites of pET15b (Novagen) to obtain plasmid pET15_EcOMP85 to express wt-BamA into cytosolic inclusion bodies. Plasmid pET15-omp85-Cys was based on pET15_EcOMP85. In plasmid pET15-omp85-Cys, the codons in the *bamA* gene for the two native cysteines C670 and C680 of BamA were replaced by codons for alanine. Both plasmids were purchased from Trenzyme (Konstanz, Germany). Plasmid pET15-omp85-Cys served as a template to prepare eight new plasmids encoding single-cysteine mutants of BamA, named X*n*C-BamA, where X represents the amino acid residue replaced by cysteine and *n* its position in the amino acid sequence of BamA. For site-directed mutagenesis, *E. coli* XL10 Gold cells (TetrD(*mcrA*)*183* D(*mcrCB-hsdSMR-mrr*)*173 endA1 supE44 thi-1 recA1 gyrA96 relA1 lac* Hte [F’ *proAB lacI*q*Z*D*M15* Tn*10* (Tet^r^) Amy Cam^r^]) were transformed with pET15-omp85-Cys. Mutageneses were performed using the QuikChange kit (Agilent Technologies, Santa Clara, CA, USA) and the PCR primers (purchased from Eurofins Genomics, Ebersberg, Germany) listed in Table S1 of the supporting information. All PCR reactions were performed utilizing Pfu Ultra polymerase (Agilent Technologies, Santa Clara, USA). Plasmids for the expression of R572C-BamA, V573C-BamA, N574C-BamA, L575C-BamA, T576C-BamA, G577C-BamA, K578C-BamA, and V579C-BamA were prepared. Each plasmid was sequenced (GATC, Konstanz, Germany) to confirm the presence of the encoding X*n*C-*bamA* gene.

For the expression of X*n*C BamA, *E. coli* strain BL21(DE3) omp8 fhuA [F^–^, *ompT hsdS*_B_ (r_B_^−^ m_B_^−^) *gal dcm* (DE3) Δ*lamB ompF*::Tn5 Δ*οmpA* Δ*οmpC* Δ*fhuA*] [34] was transformed with the plasmid harboring the required X*n*C-*bamA* gene. As in previous work on OmpA [14], the exchange of the two native cysteines by alanines did not have any effect on the formation of inclusion bodies or the expression levels of mutants of BamA in comparison to wt-BamA.

### 2.2. Isolation of wt-BamA and XnC-BamA Mutants

Overnight cultures of *E. coli* cells expressing either a selected X*n*C-BamA mutant or wild-type BamA were used to inoculate Luria–Bertani (LB) medium (containing 0.1 g/L ampicillin) at a ratio of 1:25. The cells were grown at 37 °C to an *A*_600_ of approx. 0.6–0.8. For the overexpression of X*n*C-BamA or wt-BamA, IPTG was then added to a final concentration of 0.2 mM. After 3 h, the cells were harvested by centrifugation for 15 min at 5000× *g* at 4 °C. The cells were then resuspended in Tris buffer A (20 mM Tris, pH 8.0), and lysozyme was added to a final concentration of 50 μg/mL. The mixture was stirred for 30 min at RT and then sonicated for 30 min using the macrotip of a W-450D Branson ultrasonifier (at 20% power and at 50% pulse cycle) while being cooled with an ice/water bath. The cells were then centrifuged at 8000× *g* for 20 min at 4 °C. The supernatant was removed, and the pellet was resuspended in Tris buffer B (20 mM Tris, pH 8.5, containing 8 M urea and 0.1% *β*-mercaptoethanol). The suspension was centrifuged at 3000× *g* for 30 min at RT. The supernatant was loaded onto a Q-Sepharose FF column (GE Healthcare Life Science, Freiburg, Germany), and BamA was eluted by applying a gradient from 0 to 500 mM NaCl in Tris buffer B. Fractions containing BamA were pooled and concentrated. The concentration of BamA was determined as described [35]. The typical yields of BamA were ~60 mg/L of the cell culture.

### 2.3. Preparation of Lipid Bilayers

1,2-dilauroyl-*sn*-glycero-3-phosphocholine (*di*C_12_PC) was purchased in powder form from Avanti Polar Lipids (Alabaster, AL, USA) and was dissolved in a mixture of chloroform and methanol (1:1). Thin films of *di*C_12_PC were prepared by removing the solvent with a stream of nitrogen and subsequent drying under vacuum for 4 h in a desiccator. The lipid films were hydrated in borate buffer (10 mM sodium tetraborate, pH 10), followed by seven cycles of freezing in liquid nitrogen and thawing at 45 °C in a water bath. To prepare LUVs, lipid vesicles were extruded 30 times through a polycarbonate membrane with a pore diameter of 100 nm (Nucleopore, Whatman/Cytiva, Clifton, NJ, USA) using a mini-extruder (Avanti Polar Lipids, Alabaster, AL, USA). This method results in monodisperse vesicles with a size distribution of diameters of ~100 ± 30 nm; for details see, e.g., refs. [36,37]. Within this size distribution, the kinetics of the folding and insertion of another outer membrane protein (OmpA) were largely unaffected, at least for vesicle diameters between 50 and 100 nm [18]. The vesicles are stable for at least 1 day. LUVs were used for folding experiments on the day of preparation.

### 2.4. Labeling of Cysteine Residues

Each X*n*C-BamA was labeled with 5-((((2-Iodoacetyl) amino) ethyl) amino) naphthalene-1-sulfonic acid (IAEDANS), as described [38]. Briefly, an isolated X*n*C-BamA mutant was diluted in Tris buffer (20 mM Tris, pH 7.2), containing 2 mM EDTA and 7 M urea to a final concentration of approx. 50 μM. The thiol group of X*n*C-BamA was then reduced at a fivefold molar excess of TCEP while flushing the sample with nitrogen gas. After an incubation for 30 min at RT, the labeling process was started by the addition of a 10-fold molar excess of IAEDANS and further incubation for at least 12 h at RT. To remove the excess of labeling reagents, the sample was dialyzed against 300 mL of Tris buffer (20 mM Tris, pH 8.5, containing 2 mM EDTA). The buffer was replaced seven times. The labeled X*n*C-BamA was concentrated by centrifugation using Amicon Ultra-4 concentrators (Merck KGaA, Darmstadt, Germany) with a 10 kDa molecular mass cut-off. The concentration of the labeled X*n*C-BamA mutant was then determined [35]. All mutants were fully labeled, which was confirmed with 5,5′-dithiobis (2-nitrobenzoicacid) (DTNB, “Ellman’s reagent”), as described [38,39,40].

### 2.5. Folding of BamA and Trypsin Digestion

The folding of BamA in LUVs composed of *di*C_12_PC was initiated by a strong dilution of the unfolded BamA (from a stock solution in borate buffer containing 8 M urea) into preformed *di*C_12_PC bilayers in borate buffer, diluting the urea ~20-fold. The final concentrations in the folding reaction mixtures were 7 μM BamA and 7 mM *di*C_12_PC. Folding was initiated either at 2 °C to examine a trapped folding intermediate or at 40 °C to obtain folded BamA. To determine the progress of the folding and insertion into lipid bilayers, different incubation times were selected. For analysis, BamA (10 μM) was hydrolyzed by the addition of 1 μg of trypsin per 50 μg of BamA for three hours at 37 °C. For analysis by SDS-PAGE, 4 μL of 5× Laemmli Buffer was added to 16 μL of each sample at a final volume of 20 μL. These samples were then loaded onto a 12% acrylamide, N,N-methylenbisacrylamide gel. Undigested wt-BamA and X*n*C-BamA mutants were also loaded on the gels, but at a slightly lower concentration to account for dilution with a solution of trypsin in the samples that were subject to proteolysis. As a molecular mass marker, the Page Ruler Prestained Protein Ladder from Thermofisher Scientific (Schwerte, Germany) was used. SDS-PAGE was performed as described [41,42], and the polyacrylamide gels were stained with Coomassie Blue.

### 2.6. Circular Dichroism Spectroscopy

The formation of the *β*-sheet secondary structure of BamA was examined by circular dichroism (CD) spectroscopy. BamA (10 μΜ) was folded and inserted into bilayers of *di*C_12_PC by a 20-fold dilution of the urea and at a ratio of 1000 *di*C_12_PC/BamA. The samples were then incubated at 40 °C for at least 12 h. Urea strongly absorbs UV light at wavelengths below ~210 nm and interferes with the CD signal. It was therefore removed by dialysis against 0.5 L borate buffer. The buffer was exchanged two times. Far-UV CD spectra of BamA (10 μM), inserted and folded into *di*C_12_PC (10 mM), were recorded at RT in a 0.5 mm quartz cuvette QS (Hellma, Mühlheim, Germany) on a J-815 CD spectrometer (Jasco, Germany). The spectra were scanned in the wavelength range between 260 nm and 180 nm at an increment of 0.5 nm, an integration time of 1 s, and a scan rate of 50 nm/min. A total of six scans over the entire wavelength range were automatically recorded and averaged by the software for recording the spectra, which was provided by the manufacturer. Background spectra of lipid bilayers without BamA in the buffer were subtracted. As the high concentration of the urea required for unfolding strongly absorbs below ~208 nm, leading to unreliable CD-data, the spectra of the unfolded forms have been recorded between 208 and 260 nm. To minimize the absorption by urea, unfolded BamA (50 μM in borate buffer containing 8 M urea) was placed in a quartz cuvette of a 0.1 mm pathlength. The concentration of BamA in the samples was determined [35], and the spectra were then normalized to obtain the mean residue molar ellipticity [*Θ*](*λ*) in degrees square centimeters per decimole:

[*Θ*](*λ*) = 100 · *Θ*(*λ*)/(*c* · *n* · *d*) (1)

where *Θ* (*λ*) is the recorded ellipticity in degrees at wavelength *λ*, *c* is the concentration of BamA in mol/L, *n* is the number of residues of BamA, and *d* is the pathlength in centimeters of the light beam through the sample. To determine the composition of the secondary structure of BamA, the normalized spectra were analyzed with the algorithms CONTIN [43] and CDSSTR [44] and the reference spectra from datasets 4 and 7, as provided by the DICHROWEB server (see ref. [45] and the references therein).

### 2.7. Fluorescence Spectroscopy

The samples were mixed in a fluorescence cuvette (Hellma, Mühlheim, Germany). The folding of 1 μM of unfolded BamA in a 1000-fold molar excess of *di*C_12_PC (LUVs) in borate buffer was initiated by 20-fold dilution of the denaturant urea in a final volume of 1 mL. After selected incubation times at selected temperatures, as indicated in the results section, the spectra were recorded from 400 to 650 nm, with an increment of 0.5 nm and an integration time of 50 ms. Fluorescence spectra were recorded with a SPEX Fluorolog-3 fluorometer (Horiba-Jobin-Yvon, Munich, Germany) with double monochromators in the excitation and emission pathways. The fluorescence of the IAEDANS fluorophore covalently linked to an X*n*C-BamA was excited at 336 nm. A bandpass of 2.5 nm was used for both the excitation and the emission monochromators. Six scans were averaged. For background subtraction, the spectra of the samples without BamA but of otherwise identical composition were recorded first and subtracted from the spectrum of the IAEDANS-labeled X*n*C-BamA. The intensity-weighted average fluorescence emission maxima <*λ>*, given by

$$\lambda =\frac{\sum_{\lambda }f_{\lambda }\cdot \lambda }{\sum_{\lambda }f_{\lambda }}$$

were calculated, which has the advantage that the noise typically observed for instrumentally recorded intensities *f*_λ_ at a selected wavelength *λ* is averaged over the entire wavelength range. <*λ>* is therefore of a higher accuracy than a single recorded intensity at a selected wavelength. As each folding state of an IAEDANS-labeled X*n*C-BamA mutant has a characteristic <*λ>*, the time courses of the folding of each X*n*C-BamA mutant can be obtained from the <*λ*_M_*>* of the spectra of a mixture of the various folding states of BamA. Folding kinetics were analyzed by fitting either a single-exponential (Equation (2)) or a double-exponential (Equation (3)) function to the experimental time courses of the folding of BamA.

(2) $$\langle \lambda_{M}\rangle =\langle \lambda_{0}\rangle +A\cdot \exp\left(-k\cdot t\right)$$

(3) $$\langle \lambda_{M}\rangle =\langle \lambda_{0}\rangle +A_{f}\cdot \exp\left(-k_{f}\cdot t\right)+A_{s}\cdot \exp\left(-k_{s}\cdot t\right)$$

Equation (2) describes simple single-step first-order kinetics with the rate constant *k*, while Equation (3) describes kinetics composed of a slower and a faster step, with the rate constants *k*_f_ and *k*_s_, respectively.

## 3. Results

### 3.1. All Examined Single-Cysteine Mutants of BamA Fold and Insert into Lipid Bilayers of diC_12_PC

**Figure 2:**
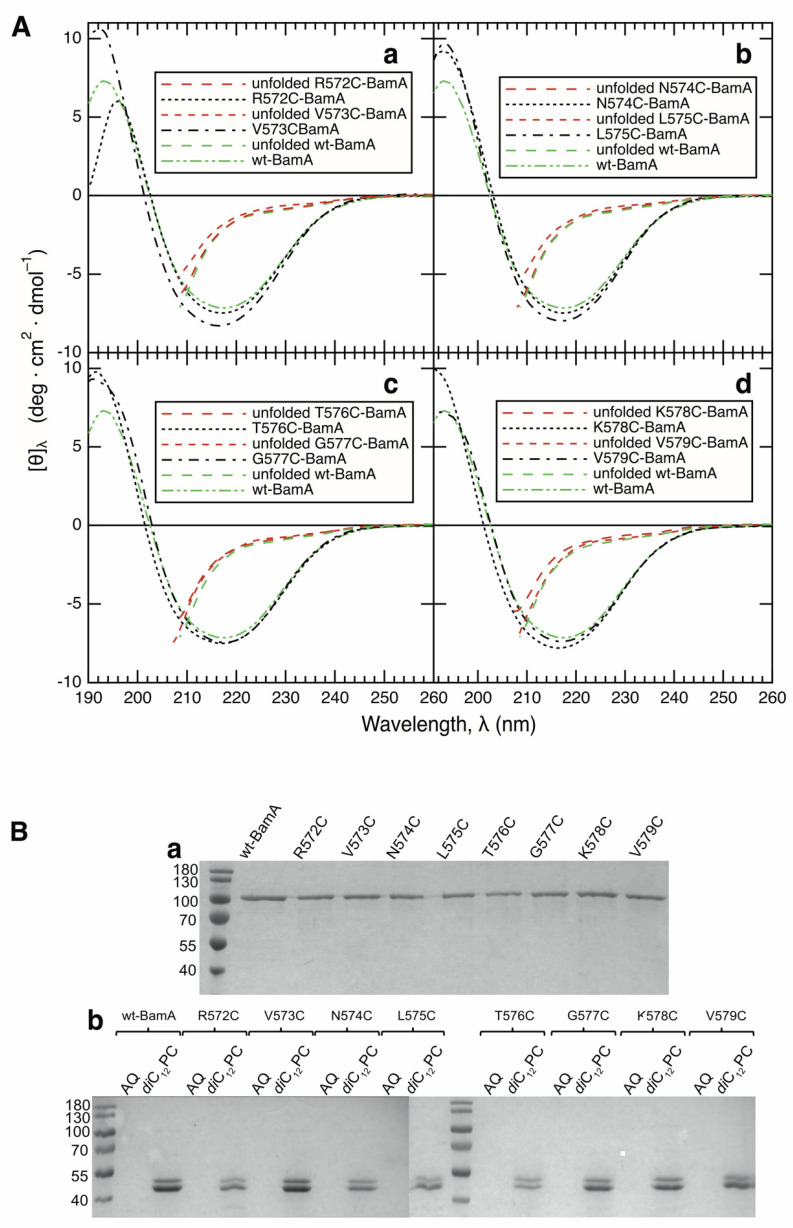
(**A**) The CD spectra of the eight single-cysteine mutants of BamA indicated their folding into native secondary structure. Each panel shows the spectra of two of the BamA mutants, (**a**) R573C- and V574C-BamA, (**b**) N574C- and L575C-BamA, (**c**) T576C- and G577C-BamA and (**d**) K578C- and V579C-BamA), either in unfolded forms (dashed lines in red) or in folded forms (dotted and dashed-dotted lines in black) in comparison to folded wt-BamA (dashed-dotted-dotted line), all in bilayers of *di*C_12_PC. (**B**) The *β*-barrel domains of wt-BamA and of the mutants of BamA insert into lipid bilayers. (**a**) Unfolded wt-BamA and unfolded X*n*C-BamA mutants isolated in buffer containing 8 M urea migrate at ~90 kDa in SDS-PAGE. (**b**) After the strong dilution of the urea in aqueous buffer (lanes 2, 4, 6, 8, 10, 13, 15, 17, 19, labeled AQ), wt-BamA and BamA mutants (10 μM) were completely hydrolyzed by the subsequent addition of trypsin. When folded into lipid bilayers, the hydrolysis of BamA by trypsin is incomplete (lanes 3, 5, 7, 9, 11, 14, 16, 18, 20, labeled *di*C_12_PC), leading to two fragments in SDS-PAGE [42] observed at ~45 kDa and at ~50 kDa.

BamA is composed of a 16-stranded *β*-barrel domain and a similarly sized periplasmic domain (Figure 1A), which fold independently from one another [46,47]. To date, the folding of the secondary structure of the transmembrane *β*-barrel domains of outer membrane proteins has been examined either by circular dichroism [7,9,15,21,23,25,27,28,48] or by infrared spectroscopy [32]. Both methods report on the overall composition of the secondary structure but do not reveal a location of the *β*-structure along the polypeptide chain. To examine the folding and insertion of BamA in regard to the formation of a transmembrane *β*-strand by cysteine scanning mutagenesis [49] and site-directed fluorescence spectroscopy [38], we expressed and isolated the single-cysteine mutants R572C-, V573C-, N574C-, L575C-, T576C-, G577C-, K578C-, and V579C-BamA in unfolded form in 8 M urea for the subsequent labeling of the reactive sulfhydryl group of the cysteine. All mutants were expressed to high yields in the form of inclusion bodies, as reported for wt-BamA [1]; an impact of the mutations on the expression yields was not observed (not shown). In the present study, the residues replaced by a cysteine were all located in strand *β*_9_ of the *β*-barrel domain of BamA (Figure 1B). In folded and inserted BamA, the even-numbered residues of *β*_9_ of the *β*-barrel domain of BamA are polar and oriented toward the barrel-lumen, whereas the odd-numbered residues of *β*_9_ are hydrophobic and face the lipids of the bilayer (Figure 1C). We first investigated whether the X*n*C-BamA mutants fold and insert into preformed bilayers of *di*C_12_PC, as described previously for wt-BamA [1]. The CD spectra of the unfolded and folded mutants in *di*C_12_PC are shown in Figure 2, together with the CD spectrum of folded wt-BamA. While the CD spectra of the unfolded proteins indicate a complete lack of a β-sheet or α-helical secondary structure, residual tertiary contacts, even at a concentration of 8 M urea, cannot be excluded. The line-shapes and amplitudes of all folded mutants corresponded well to the line-shape and amplitude of folded wt-BamA. The spectra of the folded BamA mutants were analyzed as described [43,44,45]. This resulted in ~52% *β*-sheet, ~16% α-helix, and ~32% unordered structure in BamA. For details, see Table S2 of the supporting information. These results corresponded well to the composition of the secondary structure of BamA from *Thermus thermophilus* in the solution [50] and also to the secondary structures calculated from the crystal structures of BamA from *E. coli* [3,51,52].

To further confirm the folding of the X*n*C-BamA mutants, we investigated their insertion into lipid bilayers of *di*C_12_PC by hydrolysis with trypsin, as described for wt-BamA [1]. When isolated in an unfolded form in a solution of 8 M urea, all of the mutants migrated similarly to unfolded wt-BamA near 90 kDa when examined by SDS-PAGE (Figure 2B(a)). Aqueous forms of all mutants of BamA, formed immediately after urea dilution, were completely cleaved by trypsin within 30 min, indicating that the X*n*C-BamA do not fold in the absence of lipid bilayers (Figure 2B(b)). When the X*n*C-BamA mutants were folded by dilution in the presence of preformed *di*C_12_PC membranes, followed by 12 h of incubation, all eight mutants and wt-BamA were cleaved into two major fragments, migrating at ∼50 kDa and at ∼45 kDa (Figure 2B), as previously observed for wt-BamA [1]. The size of the cleavage products corresponded to about half of the size of the entire BamA, which is consistent with the size of the transmembrane *β*-barrel domain. This 45 kDa fragment was protected against further trypsinolysis for at least 4 h. These experiments indicated an oriented insertion of all BamA mutants, as observed previously for OmpA [9] and wt-BamA [1] in similar proteolysis experiments. Together, CD spectroscopy and trypsinolysis indicated that all eight mutants folded and inserted into bilayers of *di*C_12_PC, which is consistent with our previous report on the folding of wt-BamA [1].

### 3.2. Fluorescence Spectra of IAEDANS-Labeled XnC-BamA Mutants Indicate BamA Folding

**Figure 3:**
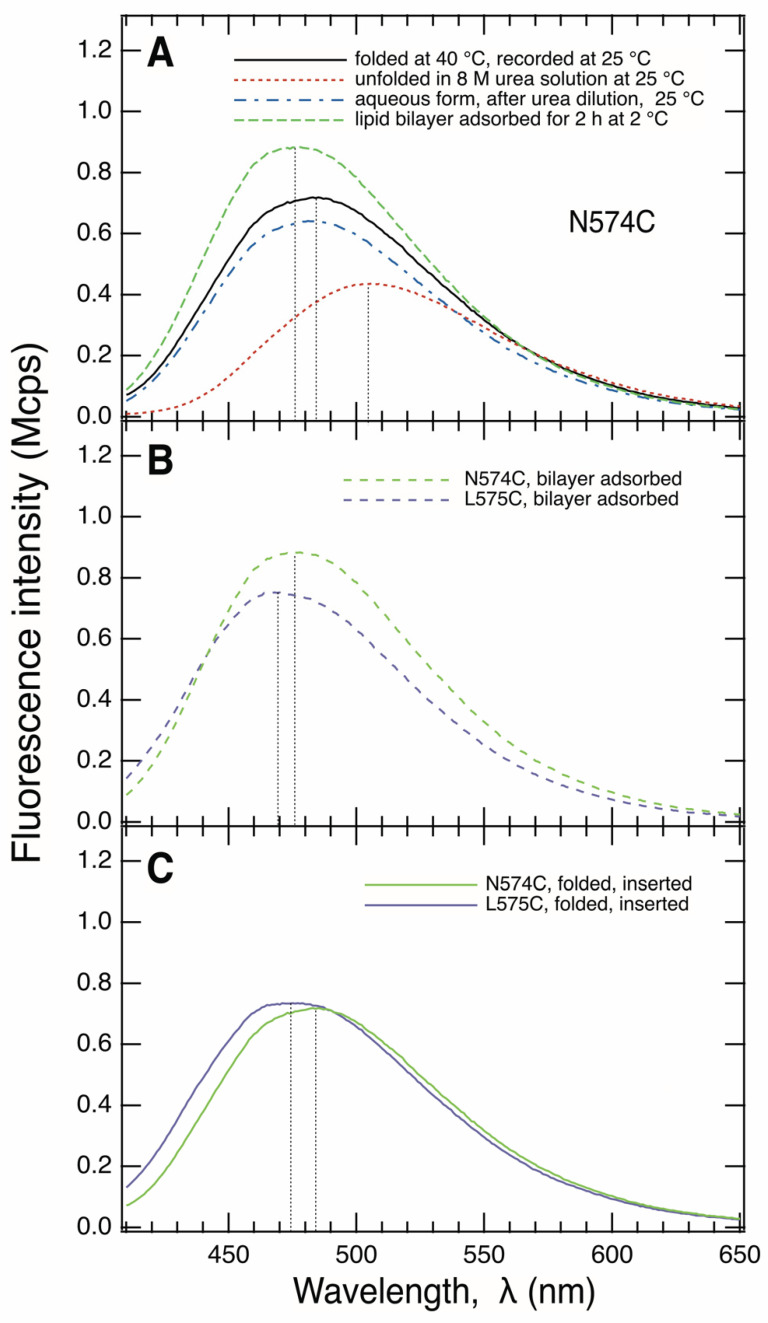
The IAEDANS-fluorescence spectra of N574C-BamA and L575C-BamA, labeled with IAEDANS at the cysteine, demonstrate an aqueous and a membrane-adsorbed folding intermediate. (**A**) Spectra of unfolded N574C-BamA in a solution of 8 M urea (dotted red line), recorded immediately after the dilution of the urea in aqueous buffer (blue dashed-dotted line), of membrane-adsorbed N574C-BamA, formed after the dilution of the urea in the presence of preformed lipid bilayers of diC_12_PC at 2 °C (green dashed line), and of membrane-inserted, folded N574C-BamA, recorded after the dilution of the urea in the presence of preformed bilayers of *di*C_12_PC and an incubation period of 12 h at 40 °C (black solid line). (**B**) Spectra of N574C-BamA (green dashed line) and L575C-BamA (blue dashed line), recorded at 2 °C after incubation with preformed bilayers of *di*C_12_PC at 2 °C for 2 h. (**C**) Spectra of N574C-BamA (green solid lines) and L575C-BamA (blue solid lines), recorded at 25 °C after folding at 40 °C.

We next examined the fluorescence spectra of the IAEDANS-labeled single-cysteine mutants of BamA. Figure 3A shows the IAEDANS fluorescence spectrum of the unfolded form of N574C-BamA, with a maximum fluorescence at *λ*_max_ ~505 nm. The same *λ*_max_ was also reported for unfolded human carbonic anhydrase II, labeled with IAEDANS at a cysteine, at high concentrations of another denaturant, guanidinium chloride [53]. Similarly, all of the other seven IAEDANS-labeled XnC-BamA prepared here showed fluorescence maxima at *λ*_max_ ~505 ± 1 nm when present in unfolded form in buffer containing 8 M urea. This indicated a similar polarity of the molecular environments of the residues 572 to 579 along *β*_9_ when BamA is unfolded. After the dilution of the urea with aqueous buffer, the maximum of the spectrum of IAEDANS-labeled N574C-BamA was blue-shifted, by Δ*λ*_max_ ~–23 nm to *λ*_max_ ~482 nm, indicating a much less polar environment of the fluorophore in comparison to its unfolded form (Figure 3A).

When IAEDANS-labeled N574C-BamA was folded into bilayers of *di*C_12_PC for at least 12 h at 40 °C, its fluorescence spectrum had a maximum at 484 nm (Figure 3A). The side chains of residues at even-numbered locations of *β*_9_ such as N574 (Figure 1) are oriented toward the lumen of the *β*-barrel and are therefore water-exposed. The relatively small difference of Δ*λ*_max_ ~+2 nm observed for the fluorescence spectra of the aqueous and folded forms of IAEDANS-labeled N574C-BamA was therefore not surprising.

In comparison to the IAEDANS fluorescence spectrum obtained for its unfolded form, the spectrum of the folded form of L575C-BamA was blue-shifted by Δ*λ*_max_ ~−33 nm to 471 nm (Figure 3C). The absolute value of this blue-shift was Δ|Δλmax| ~12 nm larger than that observed for N574C-BamA, with Δ*λ*_max_ ~−21 nm. The odd-numbered residues along strand *β*_9_, like L575 of BamA, face the hydrophobic lipids, which leads to stronger shifts of the fluorescence spectra in comparison to the spectra of the unfolded forms in solutions of 8 M urea. These fluorescence results are therefore in agreement with the formation of a local *β*-sheet secondary structure. This is consistent with the observed circular dichroism spectra of the BamA mutants shown in Figure 2A, which indicate a high fraction of the *β*-sheet structure in all folded mutants. These results are also consistent with the observed insertion of BamA into lipid bilayers, as the size of the fragments observed after the hydrolysis with trypsin indicated the protection of the *β*-barrel domain by the bilayer (Figure 2B).

### 3.3. BamA Folds via a Membrane-Adsorbed Folding Intermediate

Previous work showed the eight-stranded *β*-barrel of OmpA folds and inserts into bilayers of dioleolyphosphatidylcholine (*di*C_18:1_PC) via aqueous and membrane-bound folding intermediates [14,17,21,33], as reviewed by, e.g., [12,13,54,55,56]. As a membrane-adsorbed intermediate in the folding of OmpA was first identified at lower temperatures by fluorescence spectroscopy [9,17,30,32], we examined the folding of BamA at 2 °C. At 2 °C, BamA does not fold into bilayers of *di*C_12_PC to its native conformation and was still degraded by trypsin.

When incubated with preformed bilayers of *di*C_12_PC for 2 h at 2 °C, labeled N574C-BamA showed IAEDANS-fluorescence spectra with a maximum at *λ*_max_ ~476 nm (Figure 3B). Relative to the spectrum of the unfolded form, a blue shift of Δ*λ*_max_ ~−29 nm was observed, whereas the blue shift for folded and inserted N574C-BamA was only Δ*λ*_max_ ~−21 nm (Figure 3C), i.e., the absolute value was Δ|Δλmax| 8 nm smaller. Apparently, N574C-BamA interacts with the hydrophobic region of the lipid bilayer at 2 °C and is far less exposed to the aqueous space than what is observed for folded, bilayer-inserted N574C-BamA, where it is oriented toward the lumen of the *β*-barrel. In comparison, the absolute blue shift of the spectrum of IAEDANS-labeled L575C-BamA in bilayers at 2 °C relative to the spectrum of its unfolded form in a solution of 8 M urea was even greater than the corresponding shift observed for IADANS-labeled N574C-BamA. IAEDANS-labeled L575C-BamA had a maximum fluorescence at λ_max_ ~468 nm when bound to the bilayer with Δ*λ*_max_ ~−37 nm. The larger absolute difference, Δ|Δ*λ*_max_| of ~8 nm, indicated a more hydrophobic environment at position 575 than that at position 574, suggesting the residues at these positions point towards different or opposite directions within the bilayer at 2 °C. At this temperature, the IAEDANS-spectra of both labeled mutants reflected a significantly more hydrophobic environment than that observed in the absence of lipid bilayers.

In contrast to the folding experiments performed at 40 °C (Figure 2), X*n*C-BamA was hydrolyzed by trypsin when folding experiments were performed with bilayers of *di*C_12_PC at 2 °C for ~2 h. These results, as well as the observed differences in the IAEDANS fluorescence spectra of these mutants observed at 40 °C and at 2 °C, indicated that, at 2 °C, BamA adsorbs to the lipid bilayer but does not fold or insert to a transmembrane *β*-barrel. When the temperature was raised after 2 h of incubation at 2 °C to 40 °C, followed by additional incubation time, BamA was protected against hydrolysis by trypsin, indicating a transition of the membrane-bound form observed at 2 °C to the inserted form and suggesting a membrane-adsorbed folding intermediate.

### 3.4. Strand β_9_ of BamA Forms Prior to Insertion upon Adsorption to the Lipid Bilayer

**Figure 4:**
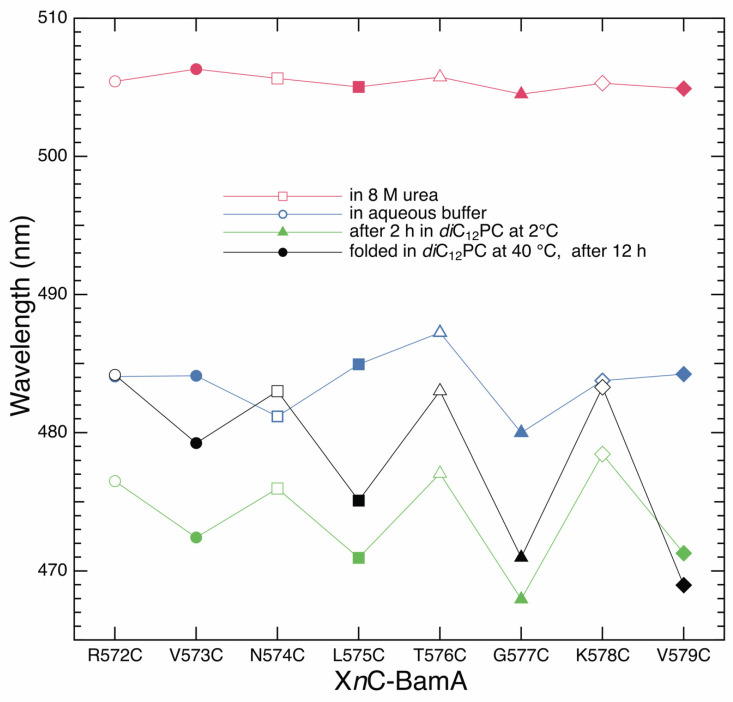
The formation of a local *β*-sheet secondary structure of strand *β*_9_ requires the adsorption of BamA into the polar/apolar interface of the membrane. The wavelength of the maximum of the fluorescence spectra of IAEDANS covalently linked to the cysteine of the X*n*C-BamA mutants is plotted as a discrete function of the position *n* of the cysteine for different folding states, namely, for unfolded X*n*C-BamA mutants in solutions of 8 M urea (red symbols), for their aqueous forms obtained immediately after the strong dilution of the urea (blue symbols), for lipid bilayer-adsorbed X*n*C-BamA at 2 °C (green symbols), and for folded inserted X*n*C-BamA obtained after 12 h of incubation at 40 °C (black symbols). Each mutant has its own symbol. Open symbols are used for mutants with an even-numbered position in strand *β*_9_, and closed symbols are used for mutants with an odd-numbered position in strand *β*_9_. Lines between data points were drawn to illustrate that the residues replaced by a single cysteine in these mutants are direct neighbors along *β*_9_. The spectra of lipid bilayer-adsorbed X*n*C-BamA were recorded at 2 °C. All other spectra were recorded at 25 °C.

For a complete analysis of the formation of strand *β*_9_ of BamA, we recorded the IAEDANS fluorescence spectra of all labeled X*n*C mutants of BamA from *n* = 572 to 579. Spectra were recorded for their unfolded forms, for their aqueous forms formed after the strong dilution of the urea in the absence of lipid bilayers, for their bilayer-adsorbed forms developed at 2 °C in the presence of preformed *di*C_12_PC membranes, and for their folded, bilayer-inserted forms. Figure 4 shows the wavelengths of the fluorescence maxima of the spectra obtained as a function of the position of the IAEDANS-labeled cysteine along strand *β*_9_. The spectra of the unfolded forms showed only minor differences in the wavelengths of the emission maxima *λ*_max_ (red). These were all located at *λ*_max_ = 506.0 ± 1 nm.

The strong dilution of the urea in the absence of lipid bilayers caused a strong shift of all fluorescence spectra toward shorter wavelengths, with the maxima located between *λ*_max_ ~479.5 and ~485.0 nm (blue). The dependence of *λ*_max_ on the position of the cysteine along the polypeptide chain was irregular for the aqueous form of BamA. However, when the BamA mutants were incubated at 2 °C with preformed lipid bilayers of *di*C_12_PC (green), the IAEDANS spectra of the X*n*C-BamA with the cysteine at an odd-numbered position showed stronger blue shifts of their fluorescence spectra, with 468 nm < *λ*_max_ < 472 nm, than those observed for the spectra of the mutants with the cysteine at an even-numbered position, which had maxima in the range 477 nm < *λ*_max_ < 479 nm. The corresponding alternating polarities of the environments of the even- and odd-numbered residues along strand *β*_9_ are consistent with the side chain orientations of an amphipathic *β*-sheet of a *β*-barrel transmembrane domain. These results suggest the local *β*-sheet secondary structure of strand *β*_9_ forms at the membrane–water interface, as BamA, which was incubated with *di*C_12_PC bilayers at 2 °C, also remained completely degradable by trypsin.

A similarly alternating polarity of the environments of even- and odd-numbered residues was also observed from the maxima of the IAEDANS fluorescence spectra of the folded and membrane-inserted X*n*C-BamA mutants (black), as expected for *β*-strands in a folded, bilayer-inserted *β*-barrel. The maxima of the IAEDANS fluorescence spectra of labeled even-numbered residues along *β*_9_ were in the range 482 nm < *λ*_max_ < 484 nm, while the maxima of the spectra of the odd-numbered residues along *β*_9_ were located in the range 468 nm < *λ*_max_ < 479.5 nm. The odd-numbered residues are expected to show stronger blue shifts of the fluorescence maxima upon folding and insertion, as they are oriented toward the hydrophobic chains of the lipids in the bilayer, while the even-numbered residues are oriented to the water-exposed lumen of the *β*-barrel.

**Figure 5:**
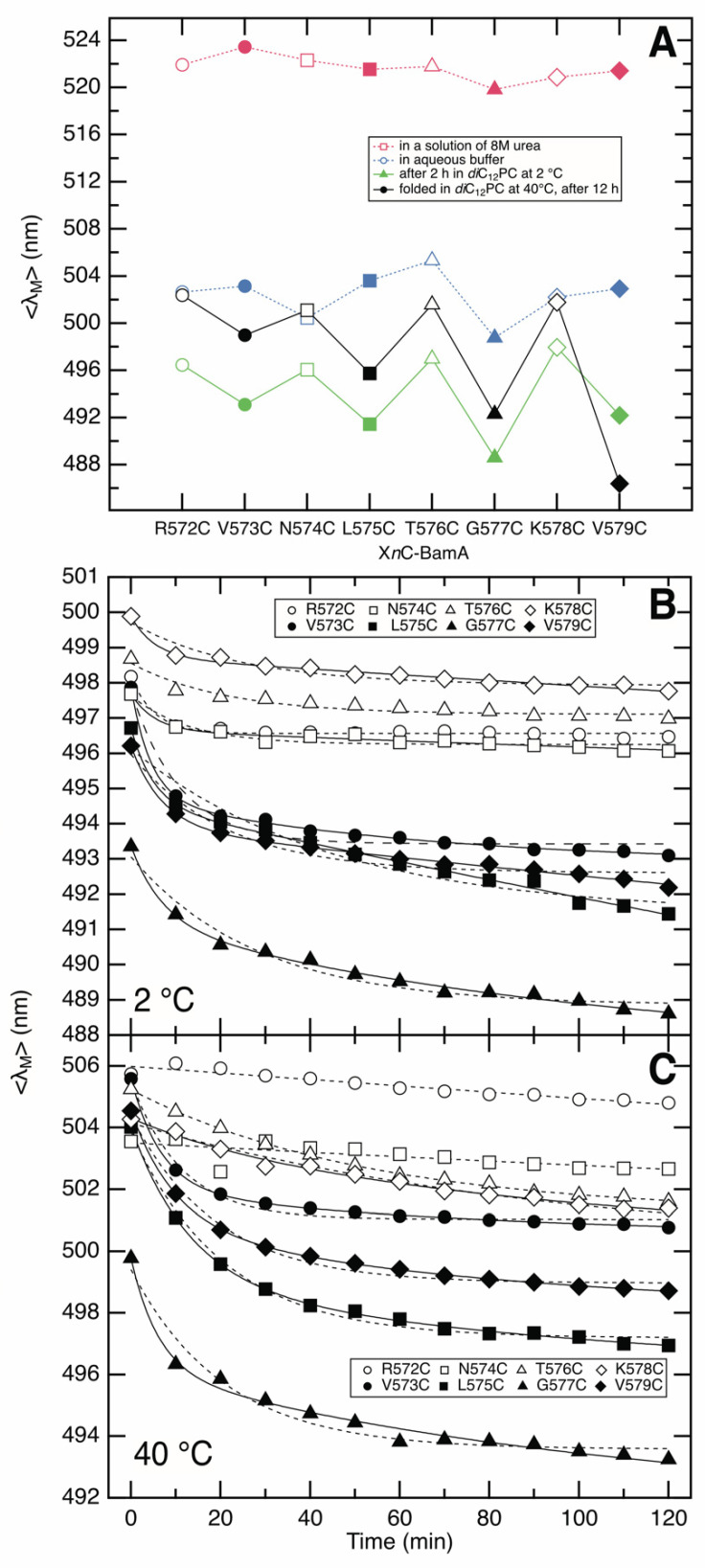
(**A**) Intensity-weighted average fluorescence emission maxima <*λ> =* Σ [*λ*·*F*(*λ*)]/Σ *F*(*λ*) of the fluorescence spectra as a discrete function of the position *n* of the cysteine. Data were calculated for unfolded X*n*C-BamA (red symbols), for the aqueous intermediates (blue symbols), for the *di*C_12_PC bilayer-adsorbed forms obtained at 2 °C (green symbols), and for the folded forms (black symbols). Each mutant has its own symbol. Open symbols are used for mutants with an even-numbered position in strand *β*_9_, and closed symbols are used for mutants with an odd-numbered position in strand *β*_9_. Lines between data points were drawn to illustrate that the residues replaced by a single cysteine in these mutants are direct neighbors along *β*_9_. Obviously, these lines should not be used for interpolations. (**B**,**C**) Time courses of <*λ>* calculated from the spectra recorded over a time course of 2 h after reacting unfolded XnC-BamA (1 μM) with preformed bilayers of *di*C_12_PC (1 mM) at 2 °C (**B**) or at 40 °C (**C**). Filled symbols denote the residues oriented toward the surface of the barrel after its folding, and open symbols denote the residues found in the lumen of the *β*-barrel after its folding (compare this to Figure 1C).

The intensity-weighted average fluorescence emission maxima <*λ> =* Σ [*λ*·*F*(*λ*)]/Σ *F*(*λ*), shown in Figure 5A as a discrete function of the position of the IAEDANS-labeled cysteine, were calculated from the fluorescence spectra obtained for unfolded X*n*C-BamA (red), for the aqueous intermediate (blue), for the form adsorbed to the *di*C_12_PC bilayer at 2 °C (green), and for the folded X*n*C-BamA (black). As anticipated, the <*λ>* of the recorded spectra depended on the examined mutant and its folding state (Figure 5A), as described above for the dependence of the absolute wavelength of the fluorescence intensity maxima of the fluorescence spectra (Figure 4). However, in contrast to the absolute maxima of fluorescence spectra, the intensity-weighted average fluorescence emission maxima correlate to the mole fractions of the folding states of each X*n*C-BamA in a folding or unfolding reaction; see, e.g., refs. [48,57,58].

### 3.5. Strand β_9_ of BamA Forms Rapidly after Adsorption to the Lipid Bilayer

To investigate the kinetics of the folding of each BamA mutant into lipid bilayers, fluorescence spectra were recorded at selected times after mixing the mutant with preformed bilayers of *di*C_12_PC. The time dependence of <*λ*_Μ_> of the recorded fluorescence spectra upon folding each X*n*C-BamA mutant was recorded at 2 °C (Figure 5B) and at 40 °C (Figure 5C). At both temperatures, the progress of <*λ*_Μ_> with time indicated that the environment of IAEDANS-labeled X*n*C-BamA mutants rapidly became less polar after mixing them with preformed *di*C_12_PC bilayers. The decrease in <*λ*_Μ_> in the presence of lipid bilayers relative to the <*λ*_Μ_> of the aqueous forms in the absence of a lipid (Figure 5A) indicated the fast adsorption of the XnC-BamA to the lipid bilayer after mixing. The residues at positions 573, 575, 577, and 579 showed a less polar environment than the residues at positions 572, 574, 576, and 578 within the first 10 min after mixing, which is consistent with a fast formation of strand *β*_9_ upon the adsorption to the lipid bilayer, as this alternating change in the polarity of the environment was not observed for the aqueous forms of the X*n*C-BamA mutants (Figure 5A). The folding kinetics of X*n*C-BamA mutants with an even number *n* were well described by single-exponential fits (dashed lines), while those of X*n*C-BamA mutants with an odd number *n* were more consistent with a double-exponential time course (solid lines), indicating two folding steps and, therefore, two different lipid bilayer-adsorbed forms of BamA. The corresponding rate constants obtained from these fits are listed in Table 1 A possible explanation for the slower folding phase observed for the odd-numbered residues may be the polarity of the environment of the lipid-facing residues changes in a subsequent step of bilayer penetration/insertion after the formation of strand *β*_9_,. This would not be observed for the even-numbered residues that remain exposed to a more polar environment and end up oriented toward the lumen of the *β*-barrel.

A comparison of the <*λ*_M_> of the spectra recorded for folded X*n*C-BamA with the <*λ*_M_> of the spectra recorded for the corresponding unfolded forms in 8 M urea suggests that the first kinetic step after urea dilution, which leads to an aqueous folding intermediate, is apparently not resolved on a time-scale from seconds to minutes. The kinetics of this folding step are apparently faster than what is recordable by our experimental setup, i.e., mixing the unfolded X*n*C-BamA with preformed lipid bilayers in a fluorescence cuvette, followed by the acquisition of fluorescence spectra.

## 4. Discussion

**Figure 6:**
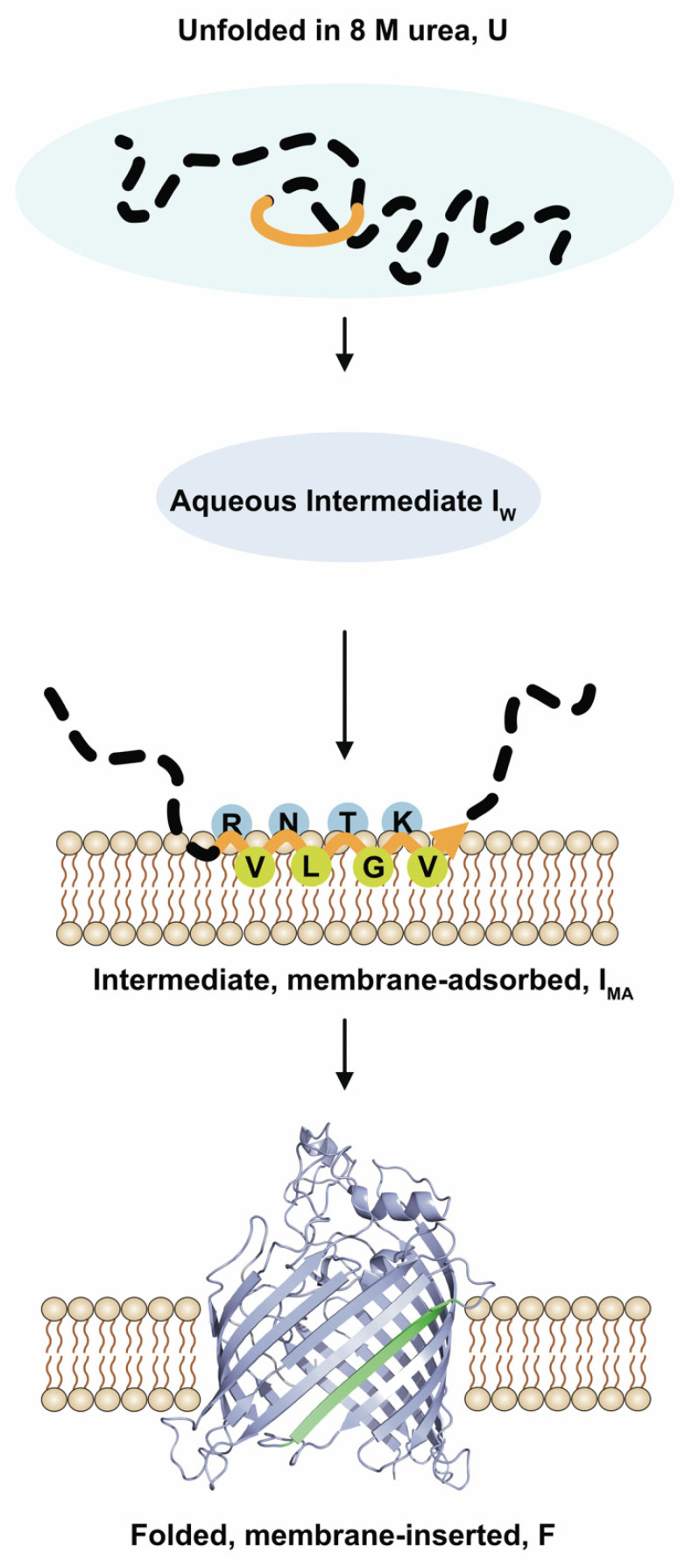
Tentative scheme for the formation of the structure of strand *β*_9_. In the first step, unfolded BamA (U) collapses hydrophobically to an aqueous intermediate (I_W_), which then adsorbs to the lipid bilayer surface and forms intermediate I_MA_. In this membrane-adsorbed intermediate, strand *β*_9_ is formed. Subsequently, BamA inserts into the lipid bilayer, forming the folded form (F). The polypeptide sequence from residues R472 to V479 is shown in orange. Parts of BamA not examined in this study are indicated as dashed black lines. In the membrane-adsorbed folding intermediate I_MA_ of BamA, the hydrophobic residues of strand *β*_9_ are located deeper in the lipid bilayer than the polar side chains.

The results of the present study are summarized in a tentative scheme of the folding and insertion of BamA into lipid bilayers (Figure 6). Upon the dilution of the denaturant urea, BamA collapses hydrophobically to form an aqueous intermediate, I_W_, as indicated by the blue shifts of the fluorescence maxima in comparison to the spectra of the unfolded form, U, of BamA. Site-directed fluorescence spectroscopy (Figure 4 and Figure 5A) suggests that, in I_W_, residues 572 to 579 along the polypeptide chain of BamA do not completely fold into a *β*-sheet structure. Instead, the secondary structure of strand *β*_9_ forms in a subsequent step when BamA adsorbs to the lipid bilayer to form intermediate I_MA_. I_MA_ can be trapped at 2 °C in bilayers of *di*C_12_PC for at least 2 h and remains accessible to trypsin-catalyzed hydrolysis. BamA folds and inserts into bilayers of *di*C_12_PC when the reaction is performed at a higher temperature—in the present study, at 40 °C.

### 4.1. BamA Folding in an Aqueous Environment

The present results indicate strand *β*_9_ of BamA does not form in the aqueous intermediate I_W_. In comparison to their unfolded forms in solutions of 8 M urea, all IAEDANS-labeled, single-cysteine X*n*C-BamA mutants show strongly blue-shifted fluorescence maxima upon urea-dilution in the absence of lipid bilayers. These shifts of Δ*λ*_max_ ≥ ~−17 nm indicate a far less polar environment of residues from R572 to V579 than that observed for the unfolded forms in 8 M urea. Similar shifts upon urea dilution in the absence of lipids or detergents were reported for the tryptophan fluorescence spectra of OmpA, with Δ*λ*_max_ ~−7 nm [30], of FomA, with Δ*λ*_max_ ~−10 nm, and of hVDAC1 [28], with Δ*λ*_max_ ~−12 nm. These shifts have been interpreted as a hydrophobic collapse of unfolded *β*-barrel proteins into an aqueous “inside-out”-intermediate upon urea dilution in the absence of a lipid or detergent [17]. It was suggested that the hydrophobic residues interact and form a more hydrophobic inside, while polar residues of the polypeptide chain would form a surface with more solvent exposure. The collapsed form would exist only transiently, would not be stable, and would tend to aggregate when lipid bilayers or detergent micelles are absent [30,33,59,60]. The *λ*_max_ (I_W,_ X*n*C-BamA) of the aqueous intermediates observed in the present study (open squares in Figure 4 and Figure 5A) may therefore represent an average of all the wavelength maxima of the fluorescence spectra of a range of coexisting dynamic states of the aqueous intermediate I_W_. Hydrogen bonds between neighboring *β*-strands are necessary to stabilize *β*-pleated sheets. The association of strand *β*_9_ with its neighbor *β*-strands would result in a conformationally rigid *β*-pleated sheet with a hydrophobic and a polar side. This would lead to differences in the *λ*_max_ of the IAEDANS-labeled X*n*C-BamA between the fluorescence spectra of IAEDANS labels on the polar side and on the hydrophobic side of the *β*-sheet. However, the IAEDANS spectra of labeled X*n*C-BamA suggest that a *β*-structure is not formed in the aqueous folding intermediate of BamA, as a clear pattern of the alternating polarity of the environment, which would indicate the formation of an amphipathic *β*-sheet upon the dilution of urea in the absence of a lipid, is not observed by fluorescence spectroscopy. In an aqueous environment, newly formed hydrophobic surfaces and intra-peptide hydrogen bonds would lead to dehydration and, eventually, the aggregation of the polypeptide chain of BamA.

The present data indicate strand *β*_9_ of BamA forms fast in the polar/hydrophobic interface of the lipid bilayer, which is consistent with the amphipathic nature of *β*-strands of transmembrane *β*-barrel domains and previous results on the formation of a *β*-structure upon the adsorption, insertion, and folding of OmpA [15], FomA [27], and PagP [61]. The residues of strand *β*_9_ facing the lumen of the *β*-barrel of BamA are either charged (R572 and K578) or polar (N574, T576) and are therefore well hydrated. On the other hand, residues facing the hydrophobic acyl chains of the lipids are mostly hydrophobic (V573, L575, V579) and favor partitioning into a hydrophobic environment [62].

### 4.2. β-Strand Formation in Polypeptide Chains of Transmembrane β-Barrel Domains Precedes Membrane Insertion for Energetic Reasons

The insertion of several peptide bonds that are not hydrogen-bonded between carbonyl and amide groups of the polypeptide backbone is unlikely for energetic reasons. The free energy of the transfer of a single non-H-bonded amide group of the polypeptide backbone from an aqueous phase to a hydrophobic phase is ~+25 kJ/mol, compared with only ~+2.5 kJ/mol for the free energy of the transfer of H-bonded peptide bonds [63,64]. For the transfer of seven peptide bonds from strand *β*_9_, the energetic cost would be ~176 kJ/mol (42 kcal/mol). It is therefore energetically very unfavorable to insert an unfolded segment of the polypeptide chain into the hydrophobic core of a membrane. The present data on the formation of strand *β*_9_ in the membrane-adsorbed folding intermediate of BamA indicate the formation of intra-peptide hydrogen bonds at the water–bilayer interface. This meets the requirement of the low energetic cost of the transfer of this strand into a transmembrane *β*-barrel structure. A small acetylated hydrophobic hexapeptide, acetyl-Trp-Leu_5_ (AcWL_5_), was previously found to be monomeric and to form a random coil structure in the aqueous phase, while about 10 to 20 of these peptides formed oligomers and a *β*-sheet structure in the bilayers of phosphatidylcholines [65]. This previous result for a hydrophobic peptide agrees well with the present data on the formation of a local structure in strand *β*_9_ of BamA.

### 4.3. Not All β-Sheets of Transmembrane β-Barrels Form in the Polar/Apolar Interface

While the present observation of the local structure formation of *β*_9_ likely applies to other *β*-strands of BamA and/or other *β*-barrel membrane proteins, it cannot be concluded that all *β*-strands of an outer membrane protein form in the membrane–water interface. The CD spectra previously obtained for FomA [27], VDAC [28], or PagP [61] after urea dilution in the absence of a lipid or detergent suggest some *β*-sheet secondary structure may form in the aqueous phase before the adsorption to the bilayer surface. For FomA, an aqueous form investigated contained a ~25% *β*-strand structure, while the folded form contained ~43%. After denaturant dilution in the absence of a lipid or detergent, VDAC (human isoform 1) contained an even larger *β*-strand structure content of 39%, which was very similar to the *β*-strand content of 36 to 39% obtained for the membrane-inserted folded VDAC in bilayers of *di*C_12_PC [28]. While circular dichroism spectra do not provide information, in which a sequence along the polypeptide chain secondary structure forms, the high amounts of *β*-strand content reported previously for aqueous forms of FomA and VDAC suggest either a rapid formation of some parts of the *β*-barrel structure upon urea dilution, even in the absence of lipid bilayers, or that some segments of the polypeptide chain form a non-native *β*-sheet structure.

### 4.4. BamA Folds and Inserts Slower Than the Smaller OmpA and Does Not Catalyze Its Own Folding

The present observations on the folding and membrane insertion of BamA into lipid bilayers can be compared to previous results on the folding and insertion of OmpA into lipid bilayers. An aqueous folding intermediate [30] as well as several membrane-adsorbed intermediates [7,14,17,21,33] have been described for the folding and insertion of OmpA into bilayers of *di*C_18:1_PC; for a review, see, e.g., [12]. However, these bilayers were of a much greater hydrophobic thickness (~27 ±1 Å) than those of *di*C_12_PC (~19.5 ± 1 Å) [66]. OmpA folded and inserted around four times faster into the thinner bilayers of *di*C_12_PC (LUVs), and intermediates of OmpA were not observed when OmpA reacted with the thinner *di*C_12_PC bilayers. When monitored by fluorescence spectroscopy, the folding kinetics of OmpA into bilayers of *di*C_12_PC were well described by a single kinetic step with a rate constant of 0.024 min^−1^ [15] at 20 °C, at an OmpA concentration of 1.1 μM, and at a *di*C_12_PC concentration of ~1.1 mM. In comparison, at very similar concentrations of X*n*C-BamA (1 μM) and *di*C_12_PC (1 mM), the folding kinetics of the much larger *β*-barrels of the X*n*C-BamA mutants into *di*C_12_PC bilayers were better described by two kinetic steps of the fluorescence time courses. Unfortunately, for this reason, it is not straightforward to compare rate constants. Overall, when observed by fluorescence spectroscopy, the time courses for the folding of X*n*C-BamA mutants were on a similar time scale (min to h) as that observed for the fluorescence time courses of OmpA interactions with *di*C_12_PC at 20 °C. For OmpA and BamA, membrane adsorption observed by fluorescence spectroscopy is not the rate-limiting step, as the folding and insertion examined by electrophoresis were slower for BamA, which is consistent with previous reports for OmpA [15].

In contrast, when observed by gel electrophoresis, the X*n*C-BamA mutants required about 6 to 12 h to fold at 40 °C, while OmpA folded into *di*C_12_PC within 2 h at 20 °C. This may be correlated to the size of the *β*-barrel transmembrane domain. The 8-stranded *β*-barrel of OmpA inserts faster than the 16-stranded *β*-barrel of BamA. Similarly, in a previous study, FomA, which is predicted to form a 14-stranded *β*-barrel [67,68], folded and inserted into *di*C_12_PC bilayers over a slow time course of ~8 to 24 h [27]. Apparently, more conformational changes are required for the folding of the larger *β*-barrels of BamA and FomA than for the folding of OmpA. These conformational changes are slower when the *β*-barrel structure forms in lipid bilayers that serve as model membranes. As in experiments with lipids extracted from *E. coli* [69], BamA alone does not facilitate its own folding and insertion into lipid bilayers of *di*C_12_PC, which is slower than the insertion of OmpA into bilayers of *di*C_12_PC described in ref. [15].

## 5. Conclusions

Aqueous and membrane-adsorbed intermediates were observed for the folding and insertion of the 16-stranded *β*-barrel domain of the TMP BamA into lipid bilayers of *di*C_12_PC. The folding and membrane insertion were much slower than those observed for the smaller eight-stranded TM *β*-barrel domain of OmpA but reveal similar folding intermediates. The combination of cysteine scanning mutagenesis and site-directed fluorescence labeling is shown to be a valuable tool in examining the local secondary structure formation of transmembrane proteins, as demonstrated here for the first time for the folding of an outer membrane protein from an unfolded form. The application of this method in this study shows that the local structure of strand *β*_9_ of BamA forms upon adsorption to the polar/apolar interface of the lipid bilayer. This method can be applied to also monitor the kinetics of local *β*-sheet formation.

## Figures and Tables

**Table 1:** Rate constants for the folding of IAEDANS-labeled X*n*C-BamA mutants into bilayers of *di*C_12_PC ^a^.

(**A**) 2 °C					
**Mutant ^a^**	**<*λ***_0_***>***	***A*_f_^c^**	***k*_f_^b^ (min^−1^)**	***A_f_* ^d^**	***k*_s_^b^ (min^−1^)**
R572C	496 ± 0.0	1.61 ± 0.07	0.19 ± 0.03	-	-
V573C	492.8 ± 0.2	3.1 ± 0.1	0.23 ± 0.03	1.9 ± 0.1	0.016 ± 0.004
N574C	494.0 ± 19	1.0 ± 0.2	0.19 ± 0.11	2 ± 19	0.002 ± 0.02
L575C	484 ± 8	2.0 ± 0.2	0.29 ± 0.16	10 ± 7	0.003 ± 0.003
T576C	497.1 ± 0.5		-	-	1.4 ± 0.1
G577C	488 ± 1	2.1 ± 0.4	0.14 ± 0.04	3.7 ± 0.7	0.010 ± 0.006
K578C	482 ± 238	1.16 ± 0.17	0.18 ± 0.08	16 ± 238	0.0005 ± 0.007
V579C	485 ± 17	2.3 ± 0.2	0.14 ± 0.02	8 ± 16	0.002 ± 0.004
(**B**) 40 °C					
**Mutant ^a^**	**<*λ* **_0_***>***	***A*_f_^c^**	***k*_f_^b^ (min^−1^)**	***A_f_* ^d^**	***k*_s_^b^ (min^−1^)**
R572C	500.1	-	-	5.90 ± 0.06	0.0019 ± 0.0002
V573C	500.5 ± 0.1	3.58 ± 0.09	0.148 ± 0.006	1.50 ± 0.05	0.013 ± 0.003
N574C	499.1	-	-	4.4 ± 0.2	0.0017 ± 0.0005
L575C	495.9 ± 1.2	5.0 ± 0.7	0.076 ± 0.009	3.0 ± 0.6	0.009 ± 0.008
T576C	501.2	-	-	3.99 ± 0.03	0.0191 ± 0.0003
G577C	492.0 ± 1.4	3.4 ± 0.7	0.18 ± 0.07	4.4 ± 0.9	0.011 ± 0.008
K578C	500.8	-	-	3.43 ± 0.07	0.0157 ± 0.0006
V579C	498.1 ± 0.2	3.7 ± 0.2	0.102 ± 0.005	2.74 ± 0.07	0.013 ± 0.002

^a^ Rate constants and the other fit parameters were obtained by fitting either a single- (Equation (2)) or a double- (Equation (3)) exponential function to the time courses of the folding of the X*n*C-BamA mutants shown in Figure 5. Most time courses were sufficiently described only by a double-exponential fit function, indicating two folding steps. ^b^ Rate constants of the faster (*k*_f_) and the slower (*k*_s_) folding steps. ^c^ preexponential factor *A*_f_ of the faster step. ^d^ preexponential factor *A*_s_ of the slower step.

## Data Availability

Data is contained within the article or supplementary material.

## Supplementary Materials

The following supporting information can be downloaded at: https://www.mdpi.com/article/10.3390/membranes13020247/s1, Table S1: PCR Primers used for site-directed mutagenesis; Table S2: Analysis of the CD spectra of X*n*C-BamA mutants in lipid bilayers of *di*C_12_PC.

## Abbreviations

BamA, barrel assembly machinery protein A; borate buffer, sodium tetraborate (10 mM, pH 10); CD, circular dichroism; *di*C_10_PC, 1,2-didecanoyl-sn-glycero-3-phosphocholine; *di*C_12_PC, 1,2-dilauroyl-*sn*-glycero-3-phosphocholine; *di*C_12_PE, 1,2-dilauroyl-sn-glycero-3-phosphoethanolamine; *di*C_18:1_PC, 1,2-dioleoyl-*sn*-glycero-3-phosphocholine; EDTA, ethylenediaminetetraacetic acid; FPLC, fast protein liquid chromatography; IAEDANS, 4-(2-hydroxyethyl)-1-piperazineethanesulfonic acid; 5-((((2-Iodoacetyl) amino) ethyl) amino) naphthalene-1-sulfonic acid; LUVs, large unilamellar vesicles; mcps, million counts per second; mdeg, millidegrees; OM, outer membrane; OmpA, outer membrane protein A; OMP, outer membrane protein; PAGE, polyacrylamide gel electrophoresis; SDS, sodium dodecylsulfate; TCEP, tris(2-carboxyethyl) phosphine hydrochloride; TMP, transmembrane protein; Tris, tris(hydroxymethyl)aminomethane; wt, wild-type.
