# Supplementary material for: The Formation of β-Strand Nine (β9) in the Folding and Insertion of BamA from an Unfolded Form into Lipid Bilayers
Source: Membranes (Basel). 2023 Feb 19;13(2):247. doi: 10.3390/membranes13020247 (PMC9964827; doi:10.3390/membranes13020247)
Supplement: Supplementary file 1 [file membranes-13-00247-s001.zip › membranes-2170699-supplementary.pdf]

Supplementary Information

Table S1. PCR Primers used for site-directed mutagenesis[a].

X*n*C-BamA	Amino Acid Substitution	PCR Primers[b]
R572C-BamA	R573C	Forward: 5'-CCCGACAGATGGTTCA**t**GTGTCAACCTGACCG-3'<br>Reverse: 5'-CGGTCAGGTTGACAC**a**TGAACCATCTGTCGGG-3'
V573C-BamA	V573C	Forward: 5'-CGACAGATGGTTCACGT**tg**CAACCTGACCGGTAAAG-3'<br>Reverse: 5'-CTTTACCGGTCAGGTTG**ca**ACGTGAACCATCTGTCG-3'
N574C-BamA	N574C	Forward: 5'-CAGATGGTTCACGTGTC**tg**CCTGACCGGTAAAGTGACC-3'<br>Reverse: 5'-GGTCACTTTACCGGTCAGG**ca**GACACGTGAACCATCTG-3'
L575C-BamA	L575C	Forward: 5'-GGTTCACGTGTCAAC**tgc**ACCGGTAAAGTGACCATTCC-3'<br>Reverse: 5'-GGAATGGTCACTTTACCGGT**gca**GTTGACACGTGAACC-3'
T576C -BamA	T576C	Forward: 5'-CACGTGTCAACCTG**tg**CGGTAAAGTGACCATTCCTG-3'<br>Reverse: 5'-CAGGAATGGTCACTTTACCG**ca**CAGGTTGACACGTG-3'
G577C-BamA	G577C	Forward: 5'-GTGTCAACCTGACC**t**GTAAAGTGACCATTCCTGGATC-3'<br>Reverse: 5'-GATCCAGGAATGGTCACTTTAC**a**GGTCAGGTTGACAC-3'
K578C-BamA	K578C	Forward: 5'-GTCAACCTGACCGGT**tgt**GTGACCATTCCTGGATCG-3'<br>Reverse: 5'-CGATCCAGGAATGGTCAC**aca**ACCGGTCAGGTTGAC-3'
V579C-BamA	V579C	Forward: 5’-GTCAACCTGACCGGTAAA**tgc**ACCATTCCTGGATCGG-3’<br>Reverse: 5'-CCGATCCAGGAATGGT**gca**TTTACCGGTCAGGTTGAC-3'

[a]Mismatched nucleotides for mutagenesis are in bold face.
[b]“Forward” denotes the primer complementary to the 5′–3′ DNA strand, “Reverse” the primer complementary to the 3′–5′ DNA strand.

Table S2. Analysis of the CD spectra of X*n*C-BamA mutants in lipid bilayers of *di*C_{12}PC[a]

BamA	Algorithm	Set	α-helix (%)	β-sheet (%)	β-turn (%)	Random coil (%)	NRMSD
**wt-BamA**	CONTIN	4	16	31	22	32	0.11
	CONTIN	7	16	30	20	34	0.11
	CDSSTR	4	13	33	24	31	0.01
	CDSSTR	7	15	29	21	34	0.02
**R572C-BamA**	CONTIN	4	16	29	22	32	0.17
	CONTIN	7	16	27	20	37	0.17
	CDSSTR	4	12	34	24	30	0.02
	CDSSTR	7	17	25	22	36	0.02
**V573C-BamA**	CONTIN	4	17	30	21	31	0.06
	CONTIN	7	17	30	21	33	0.06
	CDSSTR	4	15	32	24	30	0.01
	CDSSTR	7	17	29	21	33	0.02
**N574C-BamA**	CONTIN	4	16.4	31.5	20.8	31.3	0.103
	CONTIN	7	16.4	31.5	20.8	31.3	0.103
	CDSSTR	4	13	32	24	31	0.015
	CDSSTR	7	16	31	22	32	0.018
**L575C-BamA**	CONTIN	4	18	30	21	31	0.07
	CONTIN	7	18	30	21	32	0.07
	CDSSTR	4	14	30	24	31	0.02
	CDSSTR	7	17	28	22	33	0.02
**T576C-BamA**	CONTIN	4	17	31	21	31	0.08
	CONTIN	7	17	30	20	34	0.08
	CDSSTR	4	14	30	23	31	0.01
	CDSSTR	7	17	29	20	34	0.01
**G577C-BamA**	CONTIN	4	16	32	20	32	0.11
	CONTIN	7	16	33	20	31	0.11
	CDSSTR	4	12	32	24	31	0.02
	CDSSTR	7	16	30	21	32	0.03
**K578C -BamA**	CONTIN	4	16	32	21	32	0.07
	CONTIN	7	16	31	20	33	0.07
	CDSSTR	4	13	31	24	32	0.02
	CDSSTR	7	16	29	21	34	0.02
**V579C -BamA**	CONTIN	4	15	33	20	32	0.11
	CONTIN	7	15	32	20	33	0.11
	CDSSTR	4	12	33	24	32	0.02
	CDSSTR	7	16	30	22	33	0.02

[a]After folding into bilayers of *di*C_{12}PC, the compositions of the secondary structures of wt-BamA and X*n*C-BamA mutants were analyzed from CD spectra using DICHROWEB (see *ref.* [43] and references therein). For analysis, the deconvolution algorithms, CONTIN [41] and CDSSTR [42] were used. These algorithms and the two sets of reference spectra resulted in very similar compositions of the secondary structures of BamA and X*n*C-BamA mutants.
